# Draft Genome Sequence of the Extremophile *Acidithiobacillus thiooxidans* A01, Isolated from the Wastewater of a Coal Dump

**DOI:** 10.1128/genomeA.00222-14

**Published:** 2014-04-03

**Authors:** Huaqun Yin, Xian Zhang, Yili Liang, Yunhua Xiao, Jiaojiao Niu, Xueduan Liu

**Affiliations:** School of Minerals Processing and Bioengineering, Central South University, Changsha, China, and Key Laboratory of Biometallurgy, Ministry of Education, Central South University, Changsha, China

## Abstract

The draft genome of *Acidithiobacillus thiooxidans* A01 contains 3,820,158 bp, with a G+C content of 53.08% and 3,660 predicted coding sequences (CDSs). The bacterium contains a series of specific genes involved in the oxidation of elemental sulfur and reduced inorganic sulfur compounds (RISCs).

## GENOME ANNOUNCEMENT

*Acidithiobacillus thiooxidans* is an important member of the genus *Acidithiobacillus*, which inhabits extremely acidic environments and plays key roles in bioleaching processes (1, 2). This bacterium has the capability to receive electrons and obtain energy from the oxidation of elemental sulfur and reduced inorganic sulfur compounds (RISCs) (3–5). To investigate the mechanism of *A. thiooxidans* that enables it to survive and proliferate in acidic environments and to further research relevant metabolic pathways, such as sulfur metabolism, we determined the draft genome sequence of *A. thiooxidans* A01.

The strain *A. thiooxidans* A01 was isolated from the wastewater of a coal dump in Jiangxi, China. This bacterium was cultivated with 9K basic medium [3.0 g/liter (NH_4_)_2_SO_4_, 0.01 g/liter Ca(NO_3_)_2_, 0.5 g/liter MgSO_4_·7H_2_O, 0.5 g/liter K_2_HPO_4_, and 0.1 g/liter KCl], and elemental sulfur was used as the sole electron donor (30°C at 170 rpm). Genomic DNA was extracted with the TIANamp bacteria DNA kit (Tiangen, Beijing, China). Whole-genome sequencing was performed using Illumina HiSeq 2000 (Illumina, San Diego, CA). The genome sequence was assembled with SOAP*denovo* (6). The coding sequences (CDSs) were predicted using Glimmer (7). Functional annotation was performed through homologous comparison of each putative gene against the public databases, including the nonredundant (NR) protein database, KEGG, and COG using BLAST. In addition, RNAmmer (8) and tRNAscan-SE (9) were used to identify rRNAs and tRNAs, respectively.

The draft genome of *A. thiooxidans* A01 contains a total of 3,820,158 bp distributed in 213 contigs, with a G+C content of 53.08%. The annotation results revealed one 5S-16S-23S operon, 111 tRNAs, and 3,660 CDSs, of which 69% (2,537) were annotated as known proteins in the public database, 4% (136) were conserved hypothetical proteins, and 27% (987) were hypothetical proteins. Moreover, 3,361 CDSs were assigned to the metabolic pathways in KEGG groups, and 2,664 CDSs were assigned to the functional COG groups. A number of genes potentially responsible for the oxidation of elemental sulfur and RISCs were identified: a sulfide quinone reductase gene (*sqr*), a thiosulfate:quinone oxidoreductase gene (*doxD*), a tetrathionate hydrolase gene (*tetH*), a sulfur oxygenase reductase gene (*sor*), which was not detected in *A. thiooxidans* ATCC 19377 (5), and two truncated *sox* gene clusters (*resC*-*soxAX*-*resB*-*hyp*-*soxBZY* and *soxXYZA*-*hyp*-*soxB*) previously reported in *Acidithiobacillus caldus* (10). There were also several electron transport components involved in sulfur metabolism, including the NADH-quinone oxidoreductases, *bd* ubiquinol oxidases, and *bo*_*3*_ ubiquinol oxidases.

The draft genome of *A. thiooxidans* A01 provides a substantial body of information for gene prediction and functional annotation. It also provides novel insights into the metabolic versatility and adaptability of the strain under extreme acidic environments.

### Nucleotide sequence accession numbers.

This whole-genome project has been deposited at GenBank under the accession no. AZMO00000000. The 213 contigs have been deposited under accession no. AZMO01000001 to AZMO01000213.
